# Pan-caspase inhibitor F573 mitigates liver ischemia reperfusion injury in a murine model

**DOI:** 10.1371/journal.pone.0224567

**Published:** 2019-11-26

**Authors:** Mariusz Bral, Rena Pawlick, Braulio Marfil-Garza, Nidheesh Dadheech, Joshua Hefler, Aducio Thiesen, A. M. James Shapiro

**Affiliations:** 1 Department of Surgery, University of Alberta, Edmonton, Canada; 2 Department of Pathology, University of Alberta, Edmonton, Canada; National Institutes of Health, UNITED STATES

## Abstract

**Background:**

Liver ischemia reperfusion injury (IRI) remains a challenge in liver transplantation. A number of compounds have previously demonstrated efficacy in mitigating IRI. Herein, we applied three specific additive strategies to a mouse IRI screening model to determine their relative potencies in reducing such injury, with a view to future testing in a large animal and clinical *ex situ* normothermic perfusion setting: 1) F573, a pan-caspase inhibitor, 2) anti-inflammatory anakinra and etanrecept and 3) BMX-001, a mimetic of superoxide dismutase.

**Methods:**

A non-lethal liver ischemia model in mice was used. Additives in the treatment groups were given at fixed time points before induction of injury, compared to a vehicle group that received no therapeutic treatment. Mice were recovered for 6 hours following the ischemic insult, at which point blood and tissue samples were obtained. Plasma was processed for transaminase levels. Whole liver tissue samples were processed for histology, markers of apoptosis, oxidative stress, and cytokine levels.

**Results:**

In an *in vivo* murine IRI model, the F573 treatment group demonstrated statistically lower alanine aminotransferase (ALT) levels (p = 0.01), less evidence of apoptosis (p = 0.03), and lower cytokine levels compared to vehicle. The etanercept with anakinra treatment group demonstrated significantly lower cytokine levels. The BMX-001 group demonstrated significantly decreased apoptosis (p = 0.01) evident on TUNEL staining.

**Conclusions:**

The administration of pan-caspase inhibitor F573 in a murine *in vivo* model likely mitigates liver IRI based on decreased markers of cellular injury, decreased evidence of apoptosis, and improved cytokine profiles. Anakinra with etanercept, and BMX-001 did not demonstrate convincing efficacy at reducing IRI in this model, and likely need further optimization. The positive findings set rational groundwork for future translational studies of applying F573 during normothermic *ex situ* liver perfusion, with the aim of improving the quality of marginal grafts.

## Introduction

Ischemia reperfusion injury (IRI) is a well-recognized problem in liver transplantation. During the perioperative period, the liver graft is subjected to sequential insults, inexorably leading to degrees of reversible or irreversible graft damage. Such injury is then amplified upon reperfusion, when the ischemic organ comes into contact with warm, oxygenated blood, and abruptly resumes full metabolism.

Under such circumstances, progressing from ischemia to full physiologic function results in a cascade of injury, which defines IRI. This includes oxidative stress from generation of reactive oxygen species (ROS), inductional release of proinflammatory cytokines, release of damage associated molecular proteins (DAMPS), leading to caspase activation and potentially regulated or non-regulated cell death [1].

Several promising bioactive compounds have shown potential to mitigate liver IRI, and different groups worldwide have published these efforts [2–7]. In our laboratory, we previously explored the protective role of several potent molecules in minimizing ischemic injury to isolated and transplanted islets, and these experiences served as a rational basis for selecting specific compounds that held translational potential in minimizing IRI in livers. To our knowledge, none of the compounds investigated herein had previously been applied in such a setting, or had been tested as less potent, older formulations.

One strategy for minimizing IRI is the use of anti-oxidants to protect livers from oxidative stress. Based on our previous experiments with pan-caspase inhibitors, we selected F573, a highly potent inhibitor that had previously demonstrated efficacy in islet preservation [8–11]. Indeed, we had taken this approach to a small pilot randomized trial in clinical islet transplantation previously.

In a second approach, we aimed to determine the efficacy of anakinra (an IL-1 receptor agonist) and etanercept (a tumor necrosis factor alpha blocker) in the murine IRI model. The administration of these two compounds in tandem had previously demonstrated remarkable improvement for islet engraftment and metabolic function, with decreased apoptosis [12]. These findings led to the implementation of these anti-inflammatory agents in clinical practice, and indeed, at our institution, all clinical islet transplant recipients routinely receive this treatment.

In an alternate strategy, islets treated with a mimetics of superoxide dismutase had also previously demonstrated improved survival and function in culture. Within this class of compounds, metalloporphyrin analogs have demonstrated particular efficacy and we had previously shown that islets cultured in the presence of BMX-001, a powerful metalloporphyrin anti-oxidant had also demonstrated improved function and engraftment [13]. With these promising findings, we sought to investigate whether this compound could alleviate liver IRI.

Herein, using a murine *in vivo* model, we tested a rational selection of protective compounds to mitigate liver IRI. Our plan is to use the *in situ* focal liver ischemia model in mice as a screening tool to look for compounds and strategies that we could promptly translate to our large animal and clinical liver transplant trials that utilize *ex situ* normothermic preservation before transplantation, as a means to recondition injured and otherwise marginal liver grafts.

## Methods

### Study design overview

The Institutional Animal Care Committee at the University of Alberta approved the experimental protocol (AUP00002033) in accordance with guidelines established by the Canadian Council on Animal Care Organization. C57BL/6 male mice were obtained from Charles River Laboratories (Quebec, Canada).

Twenty mice were allocated to the each group in a block randomization design to minimize bias. In all groups, under general anesthesia using isofluorane, mice underwent a laparotomy followed by a non-lethal 70 percent liver hilar clamp, as previously described [14]. Liver ischemia was confirmed by observed blanching of the left and middle liver lobes. Heparin sodium (5 U), and 500 ul of normal saline were administered intra-peritoneum. Duration of ischemia was 60 minutes, followed by unclamping of the liver and confirmation of immediate reperfusion of the left and middle lobe. Midline incisions were closed followed by the application of topical lidocaine (AstaZeneca Inc, Mississauga, ON, Canada) to the incision, and a 0.1 mg/Kg dose of subcutaneous bipuvicane (Sogeval UK Limited, Sherrif Hutton, York, UK). Several previous groups had published studies indicating that the peak liver injury following partial liver clamping manifests at 6 hours, with plasma levels of elevated transaminases decreasing by approximately 24 hours, establishing this model [2, 3, 5–7].

In all treatment groups, at a two hour time point prior to liver clamping, the intervention drug was administered, dosed optimally based on previous publications. The treatments were administered as follows: 1) pan-caspase inhibitor, F573, 10 mg/Kg dose administered subcutaneously 2) Etanercept 5 mg/Kg administered intra-peritoneal, with Anakinra 100 mg/Kg administered intra-peritoneal 3) BMX-001, Maximum systemic dose with no adverse effect in mice 12 mg/Kg, given subcutaneously [10, 12, 15].

After 6 hours of recovery, general anesthesia was again induced as previously and mice were exsanguinated via cardiac puncture. Blood and liver tissue samples were obtained. Sham operations were also performed as additional controls (n = 6). Blood samples were immediately centrifuged for 2 min at 15,000 x RPM and plasma was frozen at -80°C until biochemical analysis was performed. Liver tissue samples were harvested and preserved both in 10% formalin and by flash freezing at -80°C until further analysis.

### Plasma biochemistry

Plasma biochemistry was performed using a VetTest Chemistry Analyzer (IDEXX, Westbrook, ME, USA) for levels of aspartate aminotransferase (AST), and alanine aminotransferase (ALT).

### Histology

Liver tissue samples were obtained at the termination point, and were immediately fixed in 10% formalin. Tissue was embedded in paraffin, stained with hematoxylin and eosin, and examined in a blinded fashion by an independent expert pathologist, who assigned a semi-quantitative score to evaluate for hepatocyte injury and bile sequestration. Biopsy tissue was examined for necrosis (0- absent, 1- pericentral, 2- Zone 2 and 3, 3- panlobular); hemorrhage (score 0- absent, 1- focal, 2- zonal, 3- panlobular), cholestasis (score 0- absent, 1- present); and sinusoidal dilatation (score 0- none, 1- mild, 2- moderate, 3- severe), as published previously [16].

### Markers of apoptosis

Tissue samples embedded in paraffin were stained for terminal deoxynucleotidyl transferase dUTP nick end labeling (TUNEL) using a DeadEnd Fluorometric TUNEL System (Promega, Madison, WI, USA) to assess degree of apoptosis. Since apoptosis appeared to be heterogeneously dispersed within the tissue sections, displaying patchy fields of TUNEL positive cells, whole sections of clamped liver tissue were scanned to obtain an unbiased assessment. Slides were then analyzed in blinded fashion using Olympus VS ASW Imaging software. TUNEL positive nuclei were compared to all 4’,6-diamidino-2-phenylindole (DAPI) stained nuclei for each given sample, and reported as a ratio.

Caspase-3 activity was measured by both Colorimetric Assay (R&D Systems, Minneapolis, MN, USA) and by western blot. For Colorimetric assay, tissue was lysed in cell lysis buffer and protein concentration in the supernatant was determined by Bradford Dye Binding assay. 10 mM of DTT and 100 μM of the chromophore DEVD-p nitroaniline (*p*NA) was added to each sample (200 μg protein) and incubated at 37°C for 1.5 hours followed by detection at absorbance 405 nm and cleaved caspase-3 activity was determined by comparison to the standard curve of *p*NA. For western blot, tissue lysates (30 μg protein) were separated on 10% gel for caspase-3 (ab214430, Abcam, Toronto, ON, Canada), and ß-actin (MA5-15739, Invitrogen, Grand Island, NY, USA) using SDS-PAGE electrophoresis and transferred to nitrocellulose membranes. Membranes were blocked with 5% BSA for 1 hour, prior to overnight incubation at 4°C with the primary antibody (1:2500). Secondary antibody (1:5000) was incubated for 30 minutes at room temperature and protein bands were detected by using a chemiluminscence kit (Millipore, USA) exposed on x-ray film for cleaved caspase 3 and ß-actin as control. Blot images were analyzed for densitometric calculation using ImageJ software.

### MPO and F4/80 analysis

Immune cell infiltrate was marked by F4/80 and Myeloperoxidase (MPO) staining of paraffin embedded sections. After deparaffinization, antigen retrieval was performed by Proteinase K (BioLabs, UK, 0.6U/mL) incubation for 3 minutes at 37°C in 10 mM sodium citrate buffer (pH 6.0) and microwave heated for 20 minutes. Sections were blocked (20% normal goat serum, 10% donkey serum, 1% BSA and 0.3% Triton-X) for 1.5 hours. Tissue sections were incubated overnight at 4°C with the primary antibodies, F4/80 (eBioScience, Carlsbad, CA, USA, 1:50) and MPO (Abcam Inc., Toronto, ON, Canada, 1:150), independently. Secondary antibodies labelled with Alexa Fluor 488 and 649 (Invitrogen, Eugene, OR, USA 1:500) were added for 1 hour at room temperature. Slides were mounted with Prolong Gold Antifade DAPI stain (Invitrogen, Eugene, OR, USA) and images were recorded on a Zeiss COLIBRI epi-fluoresence inverted microscope and Zen10 acquisition software.

### Cytokine and biomarker analysis

Plasma and tissue samples were maintained frozen at -80°C until both were analyzed for cytokine and biomarker content (Interleukin 1 (IL-1), Interleukin 4 (IL-4), Interleukin 5 (IL-5), Interleukin 6 (IL-6), Interleukin 10 (IL-10), Tumour necrosis factor alpha (TNF-α), Interferon gamma (IFN-γ), and KC/GRO) using Pro-inflammatory Panel (mouse) Kit (Meso Scale Discovery, Gaithersburg, MD, USA). Frozen tissue samples were lysed (50 nM Hepes, 1 mM EDTA, 150nM NaCl and 1% Triton-X) with homogenization prior to assay.

### Oxidative stress measurement

Oxidative stress was quantified by measurement of lipid peroxidation malondialdehyde (MDA) assay (Abcam Inc., Toronto, ON, Canada). Frozen tissue samples (-80°C) were weighed and lysed in MDA lysis buffer + butylated hydroxytoulene (BTH) with homogenization then frozen at -20°C until further analysis. Thiobarbituric Acid (TBA) was added to thawed samples, incubated for 60 minutes at 95°C, cooled to room temperature in an ice bath for 10 minutes followed by colorimetric detection (532 nm).

### Statistical analysis

Data are represented as means ± standard error of the means (SEM). Differences between continuous variables were compared using a Kruskall Wallis ANOVA or the Mann Whitney U-test. Overall comparison between groups was performed with a 95% confidence interval. A p-value of <0.05 was considered statistically significant and all the analysis was performed using Graphpad Prism (GraphPad Software Inc., La Jolla, CA, USA).

## Results

Mice were anesthetized, and the laparotomy and liver clamping procedure was performed without complications. All mice tolerated anesthesia well, and recovered without undue visible duress. At the end of the 6-hour recovery period, mice were euthanized as described, and blood and tissue samples were harvested and processed until further analysis. Due to limitations in tissue and plasma sample size, only a subset of mice were tested in each post-procedural analysis.

### F573 reduces transaminase levels post IRI

**Fig 1:**
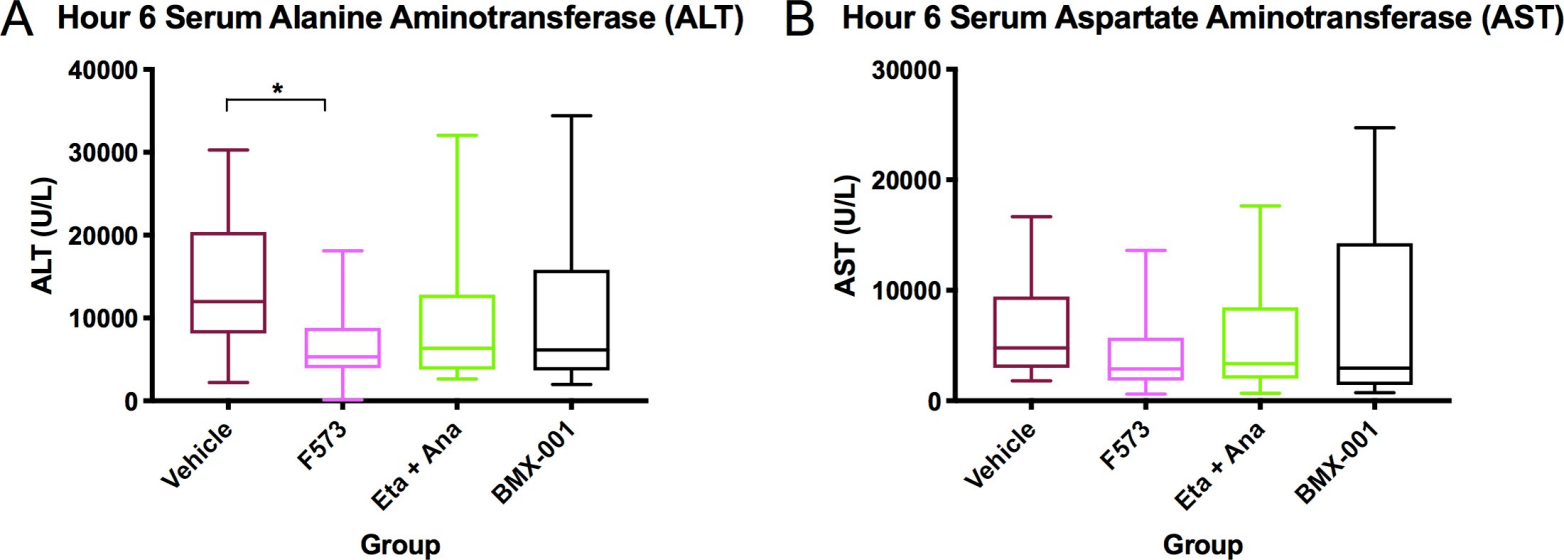
Plasma biochemistry. (A) ALT plasma levels compared to vehicle, with F573 significantly lower (p = 0.01). (B) AST plasma levels compared to vehicle (p = 0.42). Data points show means and SEM, 95% confidence interval (n = 20 per group). Aspartate aminotransferase (AST), Alanine aminotransferase (ALT).

Liver aminotransferases, aspartate aminotransferase (AST) and alanine aminotransferase (ALT) are key markers to detect liver injury. Plasma was analyzed for AST, and ALT (**Fig 1A and 1B).** All intervention groups demonstrated lower mean ALT levels with the F573 treatment group reaching statistical significance (p = 0.01) **(Fig 1A)**. AST was similarly lower in almost all treatment groups, with no statistical significance (p = 0.42) **(Fig 1B).**

### F573 reduces hepatic apoptosis

**Fig 2:**
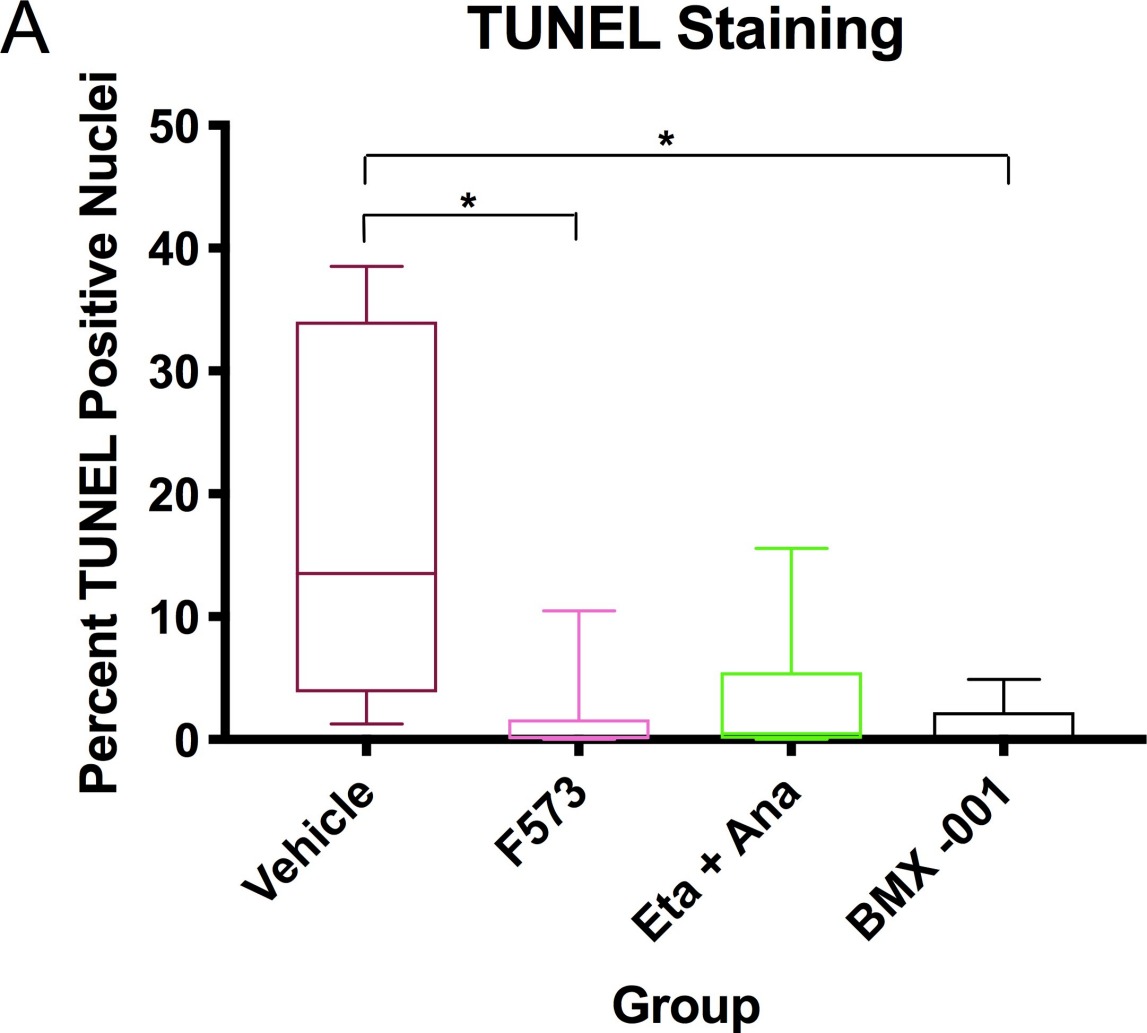
Liver tissue TUNEL staining. (A) TUNEL staining of tissue samples, reporting relative percentage of TUNEL nuclei compared to DAPI. Significantly lower TUNEL staining was found in the F573 and BMX-001 groups (p = 0.03 and p = 0.01, respectively). Data points show means and SEM, 95% confidence interval (n = 7 per group).

**Fig 3:**
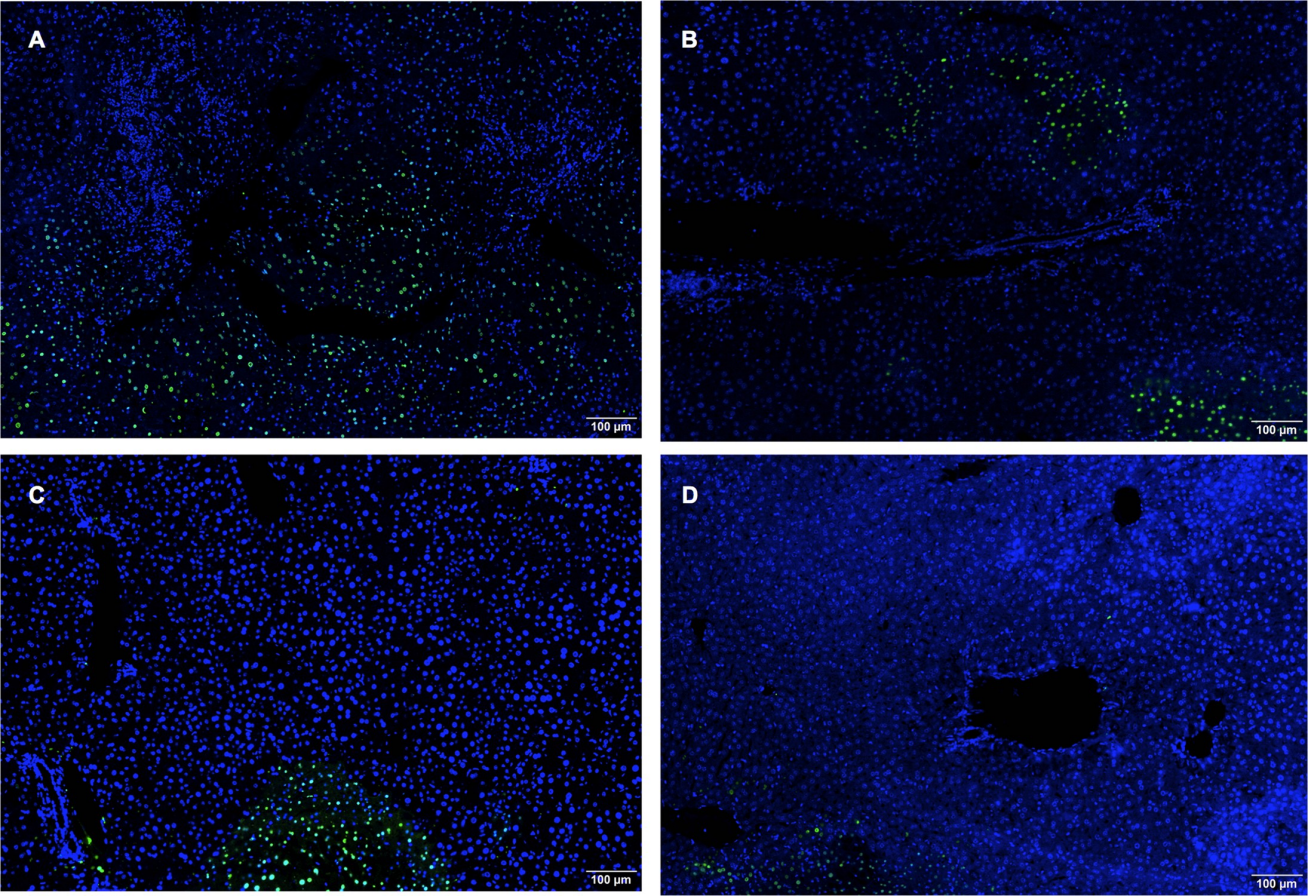
Representative sections of liver parenchyma, stained with TUNEL. (A) Representative section of tissue from a liver in the vehicle group. (B) Representative section of tissue from a liver in the F573 group. (C) Representative tissue from a liver in the anakinra and etanercept group. (D) Sample liver tissue from the BMX-001 group. Tissue was stained using fluorescein-12-dUTP dye.

Whole sections of the clamped liver tissue were stained for apoptosis by TUNEL assay, and then analyzed by Olympus VS ASW Imaging software. TUNEL staining analysis demonstrated notably reduced levels of apoptosis in all intervention groups compared to the vehicle group, with F573 and BMX-001 both reaching statistical significance (p = 0.03 and p = 0.01, respectively) (**Fig 2, Fig 3**).

**Fig 4:**
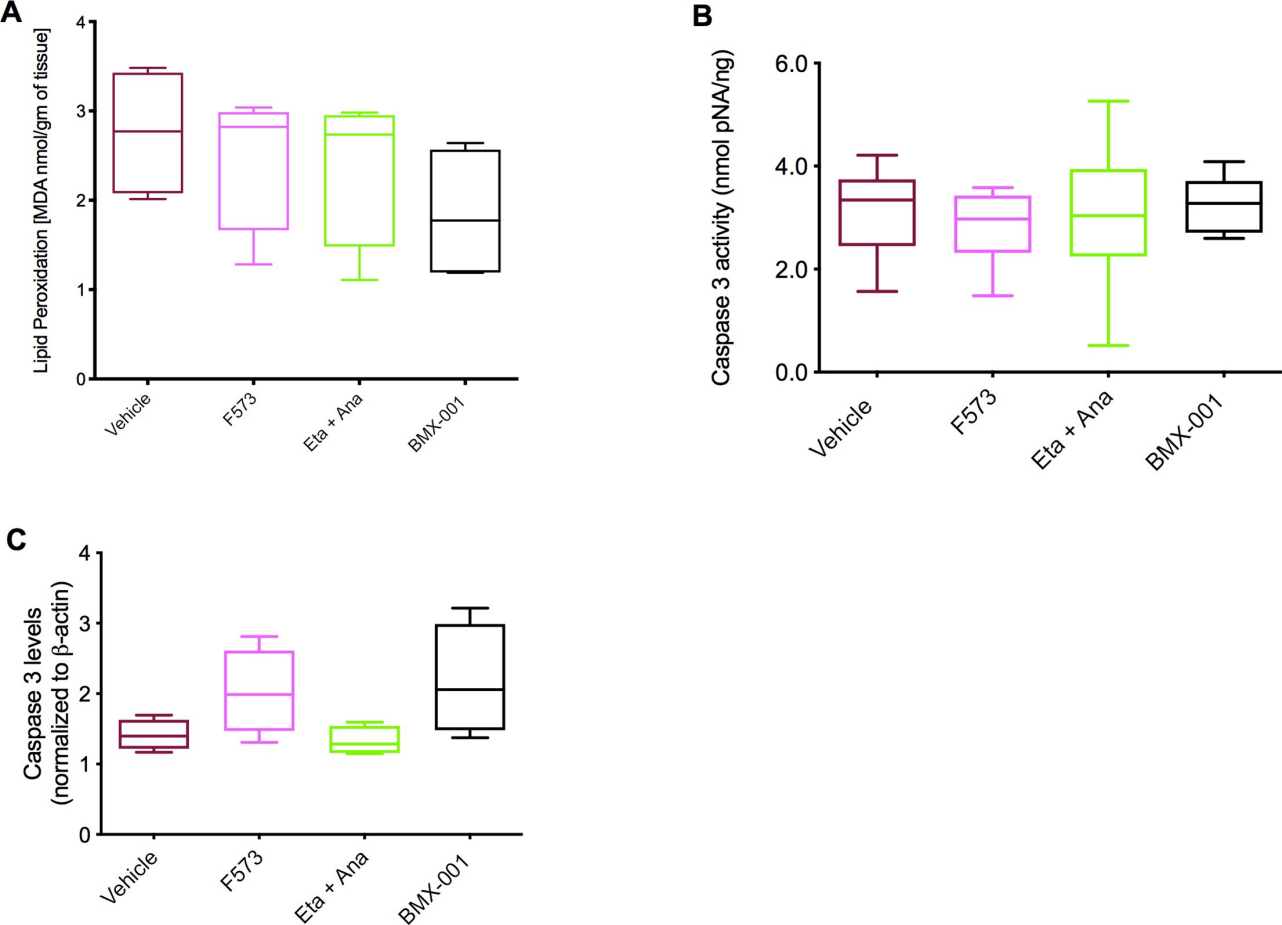
Reactive oxygen species quantification and caspase-3 activity. A. Oxidative stress quantified by lipid peroxidation malondialdehyde (MDA) assay (p = 0.45) (B) Caspase-3 activity determined by comparison to the standard curve of *p*NA (p = 0.84), (C) Caspase-3 protein level quantification normalized to ß-actin from western blot (p = 0.10). Data columns show means and SEM, 95% confidence interval (n = 4, 6, and 4, respectively per group).

Measurement of oxidative stress using a lipid peroxidation malondialdehyde (MDA) assay revealed no difference between the groups (p = 0.45, Fig 4A). There was no difference between vehicle and treatment groups when caspase-3 activity was measured by both a colorimetric assay (p = 0.84) and by western blot (p = 0.10) **(Fig 4A, 4B and 4C).**

### Neutrophil activation and macrophage sequestration

**Fig 5:**
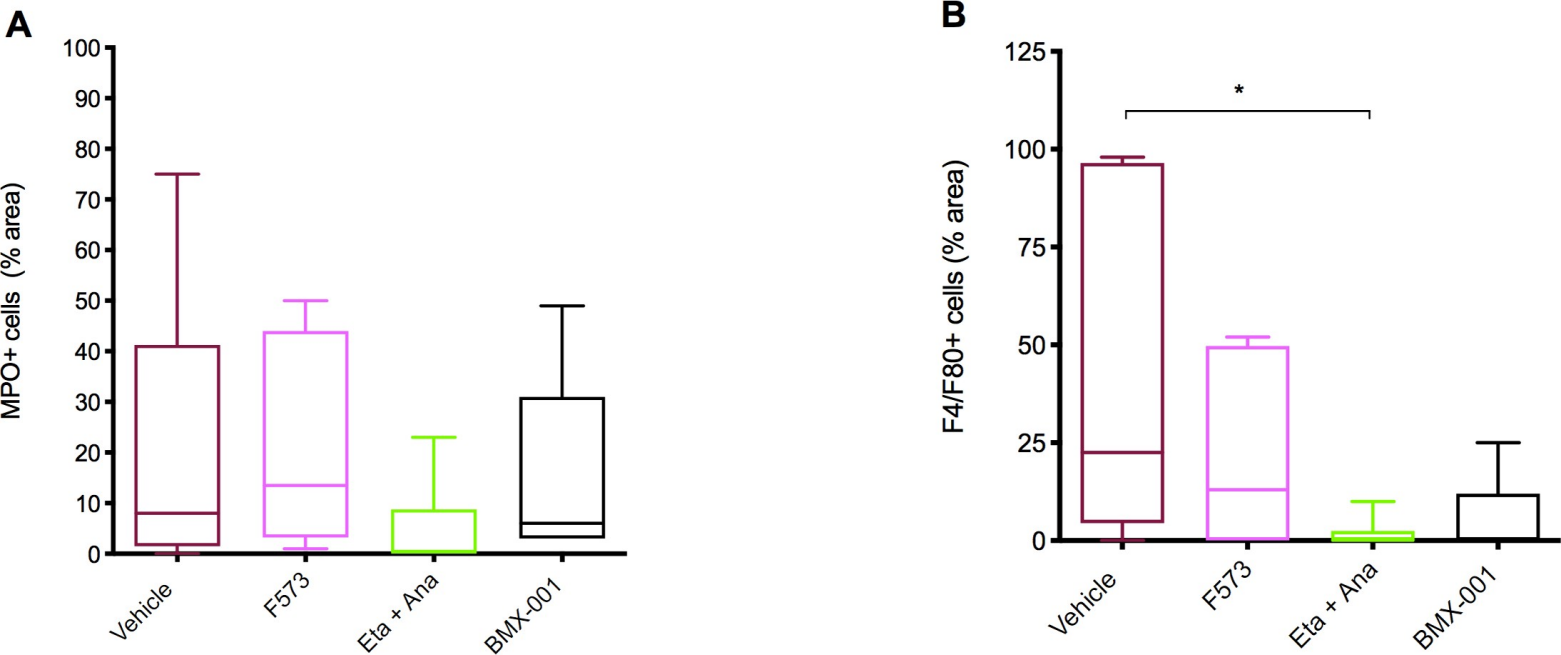
MPO and F4/80 staining. (A) MPO staining calculated as percentage of total area per tissue section (p = 0.15). (B) F4/80 staining calculated as percentage of total area per tissue section (p = 0.047). Data columns show means and SEM, 95% confidence interval (n = 5 per group).

MPO, a heme protein, allows for the detection of neutrophil infiltration and activation in the tissue. MPO staining revealed no differences between groups (p = 0.15). F4/80 antigen is expressed at high levels on macrophages, including Kupffer cells within the liver and can be used as a surrogate marker of tissue macrophage sequestration. F4/80 staining reveled a significant difference between the vehicle and anakinra/etanercept group (p = 0.047) **(Fig 5)**.

### Histological analysis

**Fig 6:**
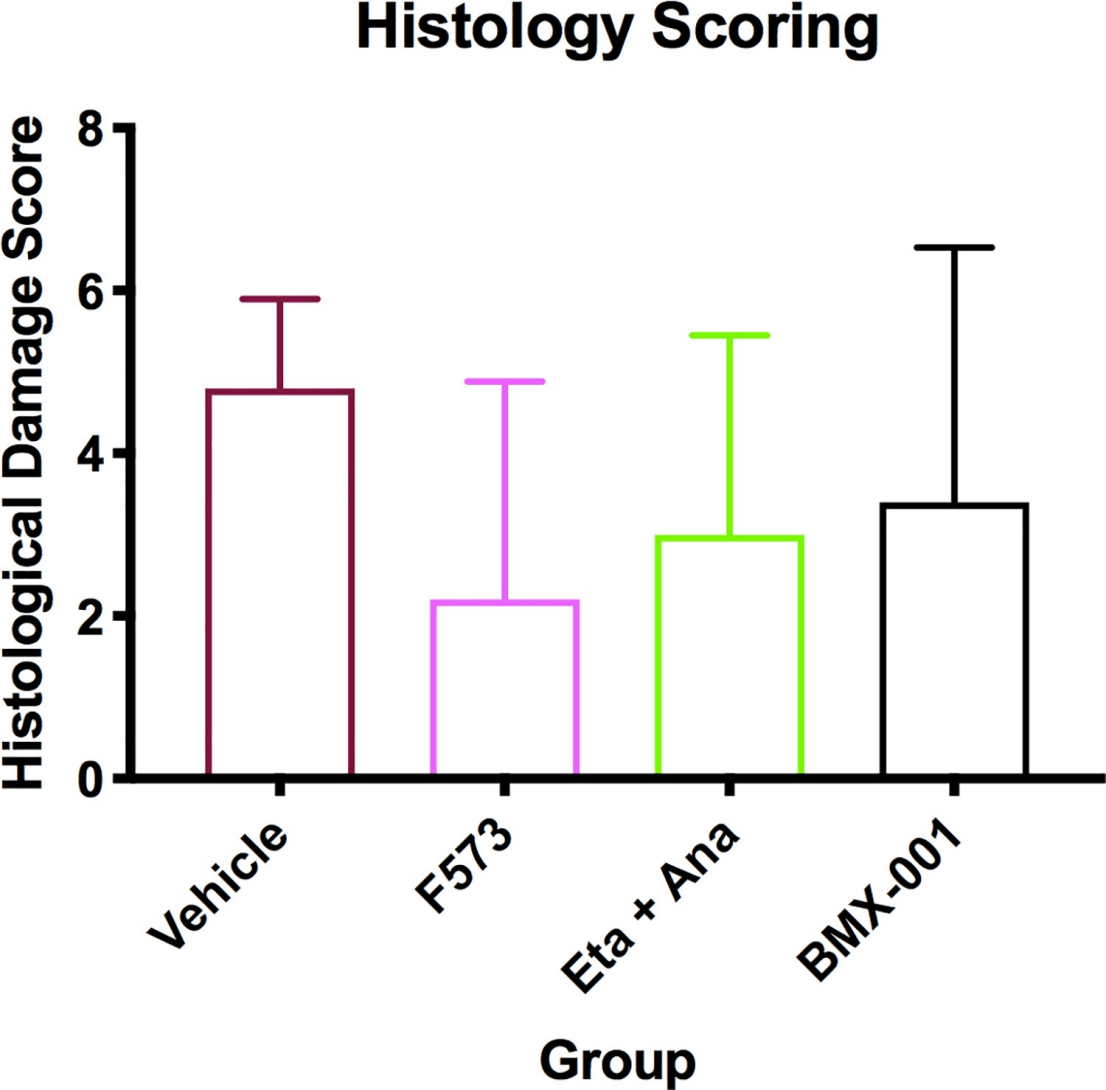
Histological scoring of liver injury. (A) All tissue slides were examined and scored by an independent expert pathologist for hemorrhage, necrosis, sinusoidal dilatation, and bile sequestration. Treated groups all demonstrated less injury, without reaching statistical significance (p = 0.11). Data columns show means and SEM, 95% confidence interval (n = 6 per group).

**Fig 7:**
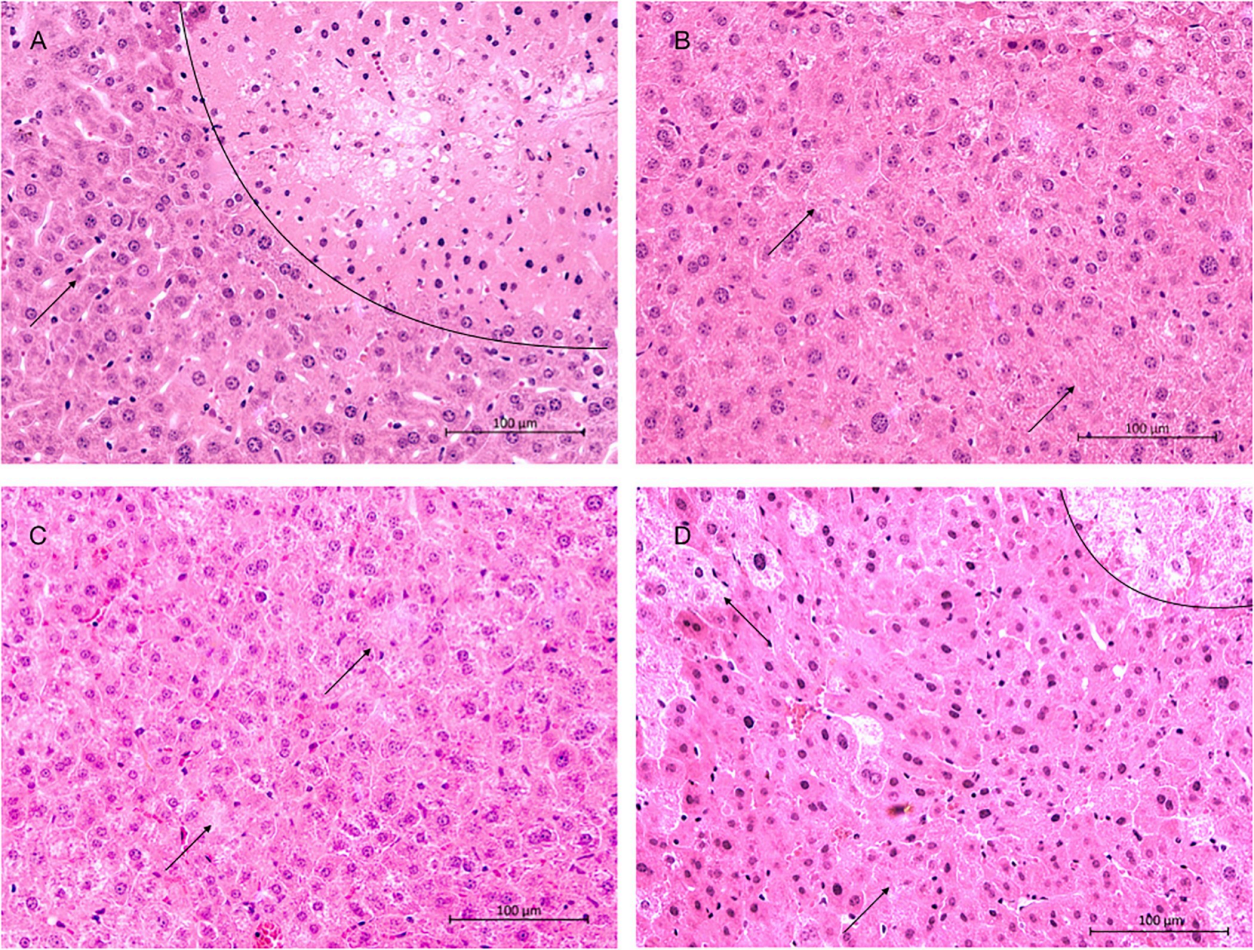
Representative histologic sections of liver parenchyma, stained with hematoxylin and eosin. (A) Representative section of tissue from a liver in the vehicle group. The curved black line indicates transition between normal tissue (left side of line) and necrotic liver (right of the line). (B) Representative section of tissue from a liver in the F573 group. (C) Representative tissue from the anakinra and etanercept group. (D) Liver tissue from the BMX-001 group. The curved black line indicates transition between normal tissue (left side of line) and necrotic liver (right of the line). All photographs are shown at 20X magnification. Arrows indicate areas of liver damage relevant to the scoring system, including necrosis and hemorrhage.

Comparison of liver histology demonstrated reduced hepatocyte injury in all treatment groups, including less hemorrhage, necrosis and sinusoidal dilatation compared to vehicle, without reaching statistical significance (p = 0.11) **(Fig 6 and Fig 7).**

### F573 reduces liver tissue cytokine profile post-IRI

**Fig 8:**
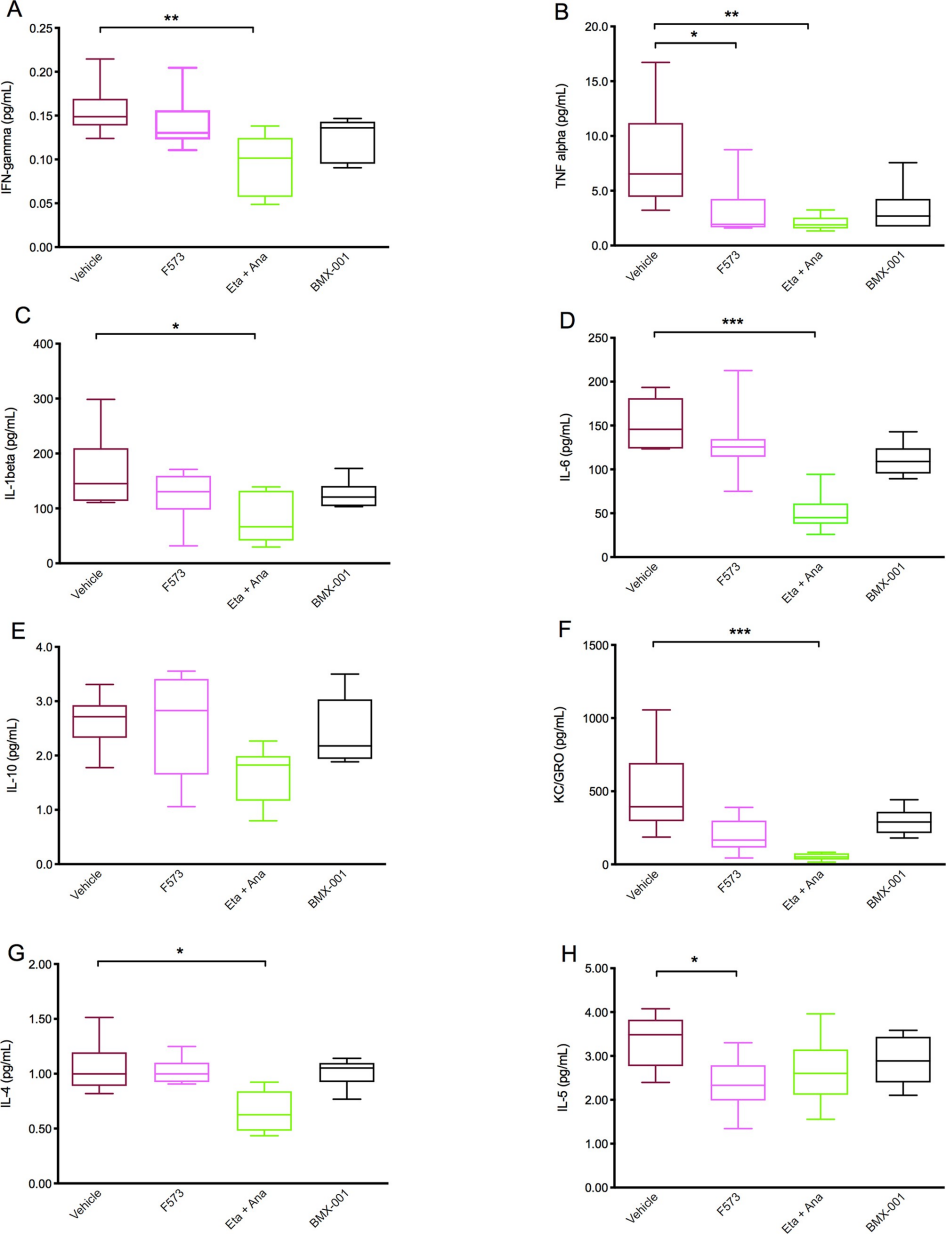
Liver cytokine and biomarker analysis. All treatment groups are compared to the vehicle group. (A) IFN-γ levels (Eta+Ana p = 0.006), (B) TNF-α levels (F573 p = 0.04); Eta+Ana p = 0.009), (C) IL-1 levels (Eta+Ana p = 0.47), (D) IL-6 levels (Eta+Ana p = 0.0003), (E) IL-10 levels (p = 0.09), (F) KC/GRO levels (Eta+Ana p = 0.0003), (G) IL-4 levels (Eta+Ana p = 0.04), (H) IL-5 levels (F573 p = 0.02). Data columns show means and SEM, 95% confidence interval (n = 6–8 per group).

Liver tissue was analyzed for cytokine and biomarker activation and compared between groups. The anakinra with etanercept group had the lowest cytokine tissue profile when compared with vehicle (IFN-γ (p = 0.006), TNF-α (p = 0.009), IL-1B (p = 0.047), KC/GRO (p = 0.0003), IL-4 (p = 0.04). The F573 group demonstrated low pro-inflammatory activity for both IL-5 (p = 0.02) and TNF- α (p = 0.04), compared to vehicle **(Fig 8)**.

## Discussion

Liver ischemia reperfusion injury is a well-known component of graft damage in liver transplantation, which potentially leads to both immediate and long-term adverse outcomes. Further, the worldwide trend of utilizing marginal and extended criteria grafts, which have a poor tolerance of cold ischemia, can lead to increased IRI events and sequelae post-transplant.

In an effort to mitigate such organ damage, different groups worldwide have administered intervening strategies, with varying success. Based on previous efficacy in islet cultivation and transplantation, we sought to determine whether a group of specifically targeted and highly potent compounds could be used to ameliorate IRI in a murine *in vivo* liver ischemia model. Should any of these additives prove to be effective in reducing such injury, this would set the stage for future application in liver *ex situ* normothermic machine perfusion (NMP).

Over the past few years, *ex situ* NMP technology has emerged as a promising platform for liver graft modification prior to transplant [17]. Such interventions have the obvious benefit of treating a graft in isolation, while minimizing or obviating any treatment side effects for the patient. Despite this potential, and the long list of possible anti-inflammatory or anti-apoptotic compounds to be added to *ex situ* perfusate, application of such interventions during NMP has not been widely published to date.

In this study, we applied three specific, selected treatments in an attempt to mitigate IRI in a murine *in vivo* model.

Pan-caspase inhibitors, a group of compounds which act on the molecular pathways leading to apoptosis, had previously been shown to reduce liver IRI [18]. F573 is a newer, more potent compound from this family, and to our knowledge had previously not been evaluated in a liver IRI model. In our laboratory, F573 had previously demonstrated reduced human and mouse islet apoptosis after *in vitro* culture, and also improved islet engraftment and significantly improved post-transplant islet function. The F573 treated group demonstrated significantly reduced apoptosis, with decreased TUNEL-positive nuclei, and decreased caspase-3 activity when compared to islets in standard culture media [10]. Further, effective pan-caspase inhibition with at least 3 alternative pan-capsase inhibitors resulted in far more effective marginal mass islet transplant engraftment in mice, including human islets, where diabetes was reversed with a 70% reduction in infused islet mass in the presence of pan-caspase inhibition [8, 9].

In our murine *in vivo* liver ischemia model, F573 also significantly reduced IRI, as evidenced by reduced ALT, as well as reduced apoptosis in TUNEL staining. Cytokine analysis revealed less pro-inflammatory TNF-α and IL-5 activity. Surprisingly, there were no significant differences noted either on caspase-3 immunoassay, MDA staining, or on western blot. We speculate that this was due to the fact that caspase-3 activation events are very short lived, and the cleaved products likely were not captured at the time of tissue collection. Activation of caspases translates rapidly to DNA damage which is ultimately captured in TUNEL staining. MPO and F4/80 staining reveled no significant differences, which we speculate results from the fact that pan-caspase inhibition would not necessarily affect neutrophil activation or macrophage sequestration. The dose administered in this study, 10 mg/Kg, had previously shown efficacy in an islet model, and was considered to be the maximal therapeutic dose [10]. Our findings suggest that such therapeutic intervention may indeed have utility in abrogating IRI, and such efficacy may be translatable into an *ex situ* machine perfusion platform.

The second intervention group was comprised of anakinra, an IL-1 receptor agonist, and etanercept, an anti-TNF-alpha agonist. Anakinra, specifically had previously demonstrated efficacy in reducing apoptosis after myocardial infarction in an experimental murine model [19]. Previous groups have administered anti-inflammatory agents in attempts to minimize liver IRI, with some therapeutics reaching clinical trials, and some anti-inflammatory therapeutic interventions also entering into the *ex situ* MP realm. Using a porcine model, Goldaracena *et al*. applied various anti-inflammatory agents to subnormothermic *ex situ* machine liver perfusions, including n-acetylcysteine, alprostadil, sevofluorane, and carbon monoxide. In the study, the treatment group had decreased AST and cytokines during perfusion, and lower bilirubin levels post transplant. The authors concluded that addition of anti-inflammatory agents did indeed augment warm machine perfusion [20].

In our previous experience in clinical and experimental islet transplantation, combined treatment using etanercept and anakinra in combination in a marginal islet mass transplant model led to remarkable improvement in islet engraftment, compared to single drug treated controls [12]. Treated islets demonstrated improved metabolic function, increased insulin secretion, and decreased apoptosis [12]. Such encouraging results allowed us to speculate that these compounds may in fact be effective in other injury models, such as IRI. In the current study, anakinra and etanercept did not demonstrate efficacy, with no statistical significance in either transaminases, MPO measurement, multiple apoptosis assays or histology. The significantly reduced macrophage sequestration can be explained by the anti-inflammatory effects of this treatment group, which would result in modulation of the immune response. Interestingly, despite the specificity of action of both anakinra and etanercept, cytokines were almost all decreased (IFN-γ, TNF-α, IL-1, IL-6, KC/GRO, IL-4 and IL-5) in this group. This can potentially be explained by the fact that TNF-α is one of the more potent cytokines, the activation and binding of which cascades into a myriad of metabolic inflammatory events. Inhibition of TNF-α, specifically would likely suppress these major downstream biochemical effects.

BMX-001 (MnTnBuOE-2 PyP5+ (Mn(III) meso-tetrakis-(N-b-butoxyethylpyridinium-2-yl) porhirin)) is a low molecular weight metalloporphyrin MnSOD mimetic that had previously demonstrated cyto-protective efficacy in a murine islet model. Islets cultured in BMX-001 supplemented media had improved insulin secretion, and exhibited significantly less apoptosis on TUNEL staining. Treated islets transplanted under the kidney capsule at a marginal dose manifested improved engraftment and function, as did human islets [13].

In this study however, the administration of BMX-001 did not abrogate IRI to a notable degree. Although the BMX-001 treated group demonstrated significantly less apoptosis as per TUNEL staining, no significant difference was seen in transaminase levels, MPO and F4/80 staining, histology or cytokine analysis. We feel that further optimization of BMX-001 would be required before utilizing the agent in an *ex situ* machine perfusion model to achieve results.

There are several limitations to our study. Due to the limited volume of liver tissue samples obtained, as well the tissue preservation method, we could only perform each analysis on a limited number of mice. In addition, TUNEL positive cells were distributed in a heterogeneously patchy fashion throughout the analyzed tissue, so each slide required scanning of approximately 15, 000 nucllei. The volume of this work restricted the number of samples we were able to process. We acknowledge the limitation in interpretation based on these smaller numbers of samples analyzed.

Further, all of the investigated interventions were administered prior to the ischemic insult, and we cannot predict at this time how this will translate to a liver *ex situ* machine perfusion setting, which would involve drug administration after injury. At the moment it is not clinically permissible to administer interventions to the donor prior to organ retrieval, which could theoretically mitigate injury upon graft reperfusion. We also cannot predict the effects of repeat drug administration, which may have additional positive effects. Considering the *in vivo* model, we are unable to predict if the additives tested have any effect on cold ischemic injury.

## Conclusions

Herein, we have shown that the pan-caspase inhibitor, F573, administered at a dose of 10 mg/Kg SC two hours before ischemic injury, mitigates IRI in a murine *in vivo* model, based on improved markers of cellular injury, decreased evidence of apoptosis, as well as improved tissue cytokine profile. Based on this positive finding, this compound can be used during *ex situ* normothermic liver perfusion, with the intention of improving marginal graft quality.

## Supporting information

S1 Experimental Dataset (XLSX)
